# Dietary Thymol Improved Growth, Body Composition, Digestive Enzyme Activities, Hematology, Immunity, Antioxidant Defense, and Resistance to *Streptococcus iniae* in the Rainbow Trout (*Oncorhynchus mykiss*)

**DOI:** 10.1155/2022/3288139

**Published:** 2022-12-30

**Authors:** Hafsan Hafsan, Marwan Mahmood Saleh, Rahman S. Zabibah, Rasha Fadhel Obaid, Hijran Sanaan Jabbar, Yasser Fakri Mustafa, Mohammed Q. Sultan, Gamal A. Gabr, Andrés Alexis Ramírez-Coronel, Mohammad Khodadadi, Mahnaz Dadras

**Affiliations:** ^1^Biology Department, Faculty of Science and Technology, Universitas Islam Negeri Alauddin Makassar, Sultan Alauddin Street, Gowa, 92118, Indonesia; ^2^College of Applied Sciences, University of Anbar, Anbar, Iraq; ^3^Medical Laboratory Technology Department, College of Medical Technology, The Islamic University, Najaf, Iraq; ^4^Department of Biomedical Engineering, Al-Mustaqbal University College, Babylon, Iraq; ^5^Department of Chemistry, College of Science, Salahaddin University, Erbil, Iraq; ^6^Department of Medical Laboratory Science, College of Health Sciences, Lebanese French University, Erbil, Iraq; ^7^Department of Pharmaceutical Chemistry, College of Pharmacy, University of Mosul, Mosul-41001, Iraq; ^8^Al-Ayen University, Thi-Qar, Iraq; ^9^Department of Pharmacology and Toxicology, College of Pharmacy, Prince Sattam Bin Abdulaziz University, Al-Kharj 11942, Saudi Arabia; ^10^Agricultural Genetic Engineering Research Institute (AGERI), Agricultural Research Center, Giza, Egypt; ^11^Universidad Católica de Cuenca, Ecuador and Universidad CES, Medellín, Colombia, Cuenca, Ecuador; ^12^Department of Fisheries, Faculty of Natural Resources, University of Tehran, Karaj, Iran

## Abstract

In this study, thymol (TYM) at dietary levels of 0, 1, 1.5, 2, and 2.5 g/kg diet was used to evaluate its effects on growth, digestive performance, immunity, and resistances to the infection induced by *Streptococcus iniae* in the rainbow trout, *Oncorhynchus mykiss*. A number of 450 fish (35.8 ± 4.4 g; Mean ± SD) were distributed to 15 tanks (30 fish/tank) in three replicates and fed TYM for 60 days. After feeding period, Fish fed 1.5-2.5 g TYM showed better growth, higher digestive enzyme activity, and body protein content compared to other diets (*P* < 0.05). Regression analysis indicated a polynomial relationship between growth parameters and dietary TYM levels. Based upon the varied growth parameters, the optimum dietary TYM level was 1.89% for FCR. TYM at dietary levels of 1.5-2.5 g significantly enhanced liver antioxidant enzyme activity [superoxide dismutase (SOD), glutathione peroxidase (GPx), and catalase (CAT)], immune components in blood [alternative complement activity (C3), total immunoglobulin (Ig), lysozyme activity, bactericidal activity, and total protein], and in mucus [alkaline phosphatase (ALP), protease activity, lysozyme activity, bactericidal activity, and total protein] compared to other diets (*P* < 0.05). TYM at dietary levels of 2-2.5 g significantly decreased malondialdehyde (MDA) levels compared to other experimental groups (*P* < 0.05). In addition, use of TYM at dietary levels of 1.5-2.5 g upregulated the expression of the immune-related genes (C3, Lyz, and Ig) (*P* < 0.05). In contrast, the expression of inflammatory genes, tumor necrosis factor (TNF-*α*) and Interleukin-8 (IL-8) significantly were downregulated in response to 2-2.5 g TYM (*P* < 0.05). The hematology of the fish also altered in response to dietary TYM, where the values of corpuscular hemoglobin concentration (MCHC), hemoglobin (Hb), red blood cell (RBC), hematocrit (Hct), and white blood cell (WBC) significantly increased in fish fed 2-2.5 g TYM compared to other diets (*P* < 0.05). In addition, MCV significantly decreased in response to 2-2.5 g TYM (*P* < 0.05). After challenge with *Streptococcus iniae*, the survival rate was significantly higher in fish fed 2-2.5 g TYM compared to other diets (*P* < 0.05). The results of the present study concluded that TYM in the diet of rainbow trout can improve the fish growth and immunity and increase the resistance of the fish to *Streptococcus iniae* infection. The results of this study recommend an optimized dietary level of 2-2.5 g TYM for the fish.

## 1. Introduction

Prevalence and spread of diseases is one of the main problems in aquaculture causing great economic losses in fish farms. Today, although a wide range of antibiotics are used to treat diseases in fish, the use of these chemicals poses health and environmental related problems. Release of antibiotics in natural water bodies can make antibiotic-resistant strains, change the natural flora and fauna, and also be biomagnified throughout food chain. In addition, antibiotics and their derivatives can accumulate in the tissues of aquatic organisms, which can be dangerous to human health as a consumer [1–3]. Therefore, use of natural materials as alternative to chemicals can be an efficient way to enhance fish immunity and to treat diseases [4, 5]. Over the last decade, the use of plant-based materials and their derivatives has increased to enhance the fish immune system [6–8]. Thymol (2-isopropyl-5-ethylphenol) (TYM), is the main phenolic compound of *Thymus vulgaris* essential oil, with antimicrobial properties [9], anti-inflammatory [10], and antioxidant [11] functions. Some studies have studied the effect of thymol as a dietary supplement on fish growth, hematology, and immunity ([12–15]). However, there is a limited data on commercial fishes such as rainbow trout with thymol.

The antioxidant system is a vital part of fish immunity since it makes the first line of enzymatic and nonenzymatic defense against free radicals [16, 17]. Although the enhancing effects of TYM on the antioxidant defense of vertebrates have been reported in many studies [14, 18–21], little is known with fish [13, 22–24]. TYM appears to stimulate the activity of the enzymes such as catalase (CAT), superoxide dismutase (SOD), glutathione (GSH), and glutathione peroxidase (GPx) [15, 25].

Cytokines are signaling metabolites that regulate immune responses in fish and other vertebrates by activating inflammatory and anti-inflammatory reactions [26]. Inflammatory responses in fish are activated by cytokines such as Interleukin-8 (IL-8) and tumor necrosis factor-*α* (TNF-*α*), while other cytokines, such as transforming growth factor-*β* (TGF-*β*) and IL-10, act as anti-inflammatory molecules and prevent extra activation of the inflammatory responses and prompt tissue recovery processes [27]. TYM has been shown to moderate inflammatory reactions [10, 28–30], however, this is somewhat unknown in fish [13]. Apart from immune components, hematological indices are also used to evaluate the overall health of fish. The hematological components reflect the health status of fish with nutritional and environmental factors and is usually assessed in nutrition studies [31]. According to literatures, TYM also affects the hematology profile [32, 33], however, this effect need to be studied more in fish [34, 35].

*Streptococcus iniae* is one of the most bacterial diseases of fish throughout the world, causing *Streptococcus* disease in the rainbow trout. This disease may result in high mortality if not controlled in the fish farms [36–38].

Considering the immunogenic properties of thymol, in the present study, the hypothesis was designed that whether thymol can have immunogenic effects in rainbow trout and protects the fish against bacterial infection caused by *Streptococcus iniae*? In this study, this hypothesis was evaluated by feeding fish with dietary levels of thymol, determining growth and immune components and resistance of fish against the bacteria.

## 2. Materials and Methods

### 2.1. Fish and Feeding Experiment

Rainbow trout (*n* = 450) with an average weight of 35.8 ± 4.4 g (Mean ± SD) were provided from a local trout farm, Karaj, Iran, transported to lab and distributed in fifteen 500 l tanks (30 fish/tank) with continuous aeration. After 14 days adaptation period, fish were fed diet containing various levels of thymol (TYM) including 0 (control), 1, 1.5, 2, and 2.5 g/kg diet in three replicates for 60 days [23]. The thymol crystals were provided from Oxford Laboratory Company, Mumbai, India with purity of 99%. Feeding was done four times a day (6 : 00 AM, 12 : 00 noon, 6 : 00 PM, and 12 : 00 midnight) to satisfaction. The experimental diets were prepared by mixing TYM with a basal diet (Table 1; crude protein: 40%, crude lipid: 15%, Ash: 9%, crude fiber: 3%, moisture: 11%, phosphorus: 1.2%, and energy content: 21.2 MJ/kg) according to [23]. The chemical composition of the basal diet in this regard, TYM was mixed with fish oil and then mixed with previously powdered basal diet. The diets were prepared in pelleted form (4 mm) and stored in plastic bags at 0°C until usage [23]. During the feeding period, Fish were under natural photoperiod, continuous flow of water was kept in tanks and water quality parameters (Mean ± SD) checked for temperature: 15.6 ± 0.3°C (Zeal thermometer, UK), oxygen: >6 mg/l (Portable Oxygen Meter, Hanna, HI9146, UK), total ammonia: <0.01 mg/l (Hi-700 Ammonia Low Range Colorimeter–Checker, Hanna instruments CO., UK), and pH: 7.3 ± 0.1 (pH meter, Hanna-HI 98128, Hanna instruments CO., UK).

### 2.2. Growth and Survival Parameters

After feeding period, the growth indices were estimated as follows:

(1) $$\begin{array}{c} \text{Weight gain percent }\left(\%\right)=\left[\text{final weight}\left(\text{g}\right)–\text{initial weight }\left(\text{g}\right)\right]/\left[\text{initial weight}\left(\text{g}\right)\right]\times 100,\\ \text{Specific growth rate }\left(\text{SGR}\right)\left(\%/\text{d}\right)=\left(\left[\ln\text{ final wt}\left(\text{g}\right)–\ln\text{ initial wt}\left(\text{g}\right)\right]/\text{days}\right)\times 100,\\ \text{Feed conversion rate }\left(\text{FCR}\right)=\text{total feed given}\left(\text{g}\right)/\text{weight gain}\left(\text{g}\right),\\ \text{Survival rate }\left(\text{SR}\right)\;\left(\%\right)=\left(\text{final numbers}/\text{initial numbers}\right)\times 100. \end{array}$$

### 2.3. Body Composition Analysis

The fish body composition of fish was determined based on the methods recommended by the Association of Official Analytical Chemists [40]. Crude protein (CP), lipid, moisture, and ash content were determined following specific procedure identification numbers of AOAC 923.03 for ash, AOAC 920.87 for protein, AOAC 945.38F; 920.39C for fat, and AOAC 925.09 for moisture. Six fish per treatment were randomly taken and then grounded by a grinder (MFW68640, Bosch, Germany). The moisture content was measured after oven-drying at 105°C to reach a constant weight. The ash content was measured through incineration of fish carcass in a muffle furnace at 550°C for 24 h. The crude protein (*N* × 6.25) and lipid content were determined by the Kjeldahl and Soxhlet methods, respectively.

### 2.4. Blood and Tissue Sampling

The samples of blood, mucus, and tissue were collected after the feeding period. Fish were first starved for 24 h and then 15 fish randomly captured from each tank. The mucus collection was conducted by putting fish in nylon bags according to Ghafarifarsani et al. [41]. The blood samples were taken from caudal vein by 2.5 ml heparinized syringe after sedation of the fish with 200 mg/l clove powder [42]. Then, the plasma was obtained through centrifugation at 13000 g for 10 min. Also, the blood slides were prepared for hematological examinations according to Kokou et al. [43]. Liver samples were collected after dissecting the fish. Finally, the plasma, mucus, and liver samples were kept in liquid nitrogen (-196°C) for further biochemical analysis.

### 2.5. Immune Parameters

The plasma and mucosal lysozyme activity was measured using lyophilized *Micrococcus luteus* according to Ellis [44]. The total immunoglobulin (Ig) concentration in plasma and mucus was assayed by polyethylene glycol method [45]. The plasma alternative complement activity (C3) was determined by measuring the haemolysis rate of rabbit red blood cells [46]. The blood bactericidal activity was determined against *Streptococcus iniae* (OD: 0.5 at 546 nm) inside a bacterial suspension and following calculating bacterial colony forming unites (CFUs) on nutrient agar plates after 24 h incubation at 35°C [47].

The total protein was determined according to Bradford [48] using a Sigma-Aldrich Protein Assay Kit. Protease activity in mucus was assayed by the Azocasein hydrolysis procedure, as described by Ross et al. [49]. Alkaline phosphatase (ALP) activity in mucus was assayed colorimetrically at 405 nm by assay kit (Sigma-Aldrich, CO, USA) based on the hydrolysis of p-nitrophenol phosphate to p-nitrophenol [49].

### 2.6. Liver Antioxidant Enzymes and Lipid Peroxidation

The activity of antioxidant enzymes in liver were assayed using commercial assay kits, according to manufacturer's instructions (Sigma-Aldrich, CO, USA). Catalase activity was assayed through the reduction of hydrogen peroxide at 240 nm [50]. Superoxide dismutase (SOD) activity was assayed through inhibiting the oxygen-dependent oxidation of adrenaline (epinephrine) to adenochrome using xanthine oxidase plus xanthine [51]. Glutathione peroxidase (GPx) activity was determined through measuring the rate of NADPH oxidation at 340 nm under glutathione reductase action [52]. The lipid peroxidation was measured spectrophotometrically at 532 nm upon reaction of malondialdehyde (MDA) with thiobarbituric acid [53].

### 2.7. Digestive Enzymes

The amylase activity was measured colorimetrically at 600 nm using a 2% starch solution (as substrate) in 0.1 M citrate phosphate buffer [54]. Lipase enzyme activity was determined at 405 nm upon hydrolysis of polyphenol myristate (as substrate) according to Gawlicka et al. [55]. Protease activity was determined at 440 nm using azocasein as substrate [56].

### 2.8. Haematological Parameters

The total number of red and white blood cells was done using a haemocytometer slide under optical microscope at 400x magnification [57]. Haematocrit (Hct) was determined by the microhematocrit method [58]. The haemoglobin (Hb) content was measured by the cyanohaemoglobin method [59]. The haematological indices [mean corpuscular volume (MCV), corpuscular hemoglobin concentration (MCHC), and mean corpuscular hemoglobin (MCH)] were estimated according to following equations [58]:

(2) $$\begin{array}{c} \text{MCHC}=\text{Hb}\times \frac{10}{\text{Hct}\;\text{MCV}}=\text{Hct}\times \frac{10}{\text{RBC}}\left(\text{million}\right)\;\text{MCH}=\text{Hb}\times \frac{10}{\text{RBC}}\left(\text{million}\right). \end{array}$$

### 2.9. Gene Expression Assay

#### 2.9.1. RNA Extraction

The total RNA content of liver tissue was extracted by acid guanidinium thiocyanate-phenolchloroform procedure according to Chomczynski and Sacchi, [60] with some modifications. The quantity and quality of the extracted RNA was estimated by determining the absorbance at 260 nm with a Nanodrop spectrophotometer (NanoDrop technologies; Wilmington, DE, USA). Also, the quality of RNA was evaluated by electrophoresis [61].

#### 2.9.2. Reverse Transcription Polymerase Chain Reaction (RT-PCR)

The extracted RNA (1 *μ*g) was used for making first-strand cDNAs by a Fermentas cDNA synthesis Kit for RT*-*PCR (Reverse transcription polymerase chain reaction). All procedures were conducted according to manufacturer's instructions by an iCycler (BioRad). The RT*-*PCR primers were synthesized based on the sequences of DNA from Gen Bank (Table 2) using Gene Runner (version 6) software. The *β*-actin gene was used as reference gene to estimate the gene expressions. The fold changes in the gene C3 (complement), lysozyme, Ig (immunoglobulin), IL-8 (Interlukin-8), and TNF-*α* (tumor necrosis factor-*α*) was measured by the 2^-*ΔΔ*Ct^ method [62]. Finally, the RT*-*PCR data were analyzed using iQ5 optical system software version 2.1 (Bio-Rad).

### 2.10. Bacterial Challenge

After 60 days feeding period, fish (10 fish/tank) were exposed to *Streptococcus iniae* by injection. The bacterium, originally isolated from diseased rainbow trout, and cultivated on agar medium for 24 h at 36°C. The dose of injection dose was selected in a previous experiment by calculation of seven-day LD_50_ (lethal dose 50), which it was 1.6 × 10^7^ cells/ml phosphate buffered saline (PBS).

The cumulative mortality of the fish was recorded daily over ten days challenge. In dead fish, the bacterial infection was confirmed by cultivation of liver tissue extract growth medium using conventional methods.

### 2.11. Statistical Analysis

The analysis of data was done using SPSS software (version 16). After evaluation of data normality by Kolmogorov*-*Smirnov test, data were subjected to One-way analysis of variance to investigate the significance. Finally, the comparison of means was conducted using Tukey test (*P* < 0.05). Also, the growth responses to dietary TYM were evaluated through polynomial regression.

## 3. Results

### 3.1. Growth Parameters

**Figure 1:**
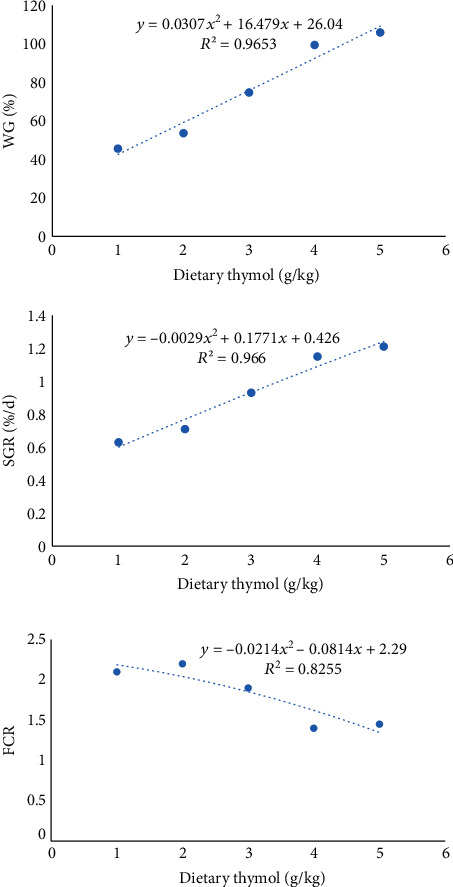
Relationships between the dietary TYM levels and growth parameters of the rainbow trout (*n* = 3).

The supplementation of fish with 1.5-2 g TYM significantly improved the growth parameters (final weight, WG (%) and SGR) in the fish compared to other diets (Table 3, *P* < 0.05). The FCR values significantly decreased in the treatments 2-2.5 g TYM compared to other experimental groups (Table 3, *P* < 0.05). There were no significant differences in growth parameters between control and the treatment, 1 g TYM (Table 3, *P* > 0.05). Also, the dose-response model (Figure 1) showed the potential response of the fish to graded levels of dietary TYM (Figure 1). In this regard, the lowest FCR was observed at 1.89% of TYM.

### 3.2. Body Composition Analysis

The protein (%) content of the fish body significantly increased in fish fed 2-2.5 g TYM compared to other diets (Table 4, *P* < 0.05). However, the lipid, ash, and moisture content had no significant differences between the experimental groups after feeding experiment (Table 4, *P* > 0.05).

### 3.3. Digestive Enzymes

The protease and lipase activities showed significant increases in fish fed 1.5-2.5 g TYM compared to control (Table 5, *P* < 0.05). The highest activity of these enzymes were observed in the group fed 2.5 g TYM (Table 5, *P* < 0.05). The amylase activity showed no significant differences between all groups after feeding period (Table 5, *P* > 0.05).

### 3.4. Plasma and Mucus Immune Parameters

The immune components in plasma (C3, total Ig, lysozyme activity, bactericidal activity, and total protein) and in mucus (ALP), protease activity, lysozyme activity, bactericidal activity, and total protein significantly increased in response to 1.5-2.5 g TYM compared to other diets (Table 6, *P* < 0.05). The highest values of immune components was observed mostly in fish fed 2-2.5 g TYM (Table 6, *P* < 0.05). There were no significant differences in immune components of plasma and mucus between control and fish fed 1 g TYM (Table 6, *P* > 0.05).

### 3.5. Hematology

The hematology of the fish altered in response to dietary TYM (Table 7, *P* < 0.05). The values of corpuscular hemoglobin concentration (MCHC), hemoglobin (Hb), red blood cell (RBC), hematocrit (Hct), and white blood cell (WBC) significantly increased in fish fed 2-2.5 g TYM compared to other diets (Table 7, *P* < 0.05). The values of MCV significantly increased in response to 1.5 g TYM compared to other diets (Table 7, *P* < 0.05). In addition, MCV significantly decreased in fish fed 2-2.5 g TYM (Table 7, *P* < 0.05). There were no significant differences in MCH between all groups (Table 7, *P* > 0.05).

### 3.6. Liver Antioxidant Enzymes

The liver antioxidant enzymes showed significant changes in response to TYM (Table 8, *P* < 0.05). In comparison with control group, the CAT activity with maximum activity in the treatments, 2 and 2.5 g TYM significantly increased in all TYM supplemented fish, while GPx and SOD elevated only in fish fed 1.5-2.5 g TYM (Table 8, *P* < 0.05). There were no significant differences in GPx and SOD activity between control and fish supplemented with 1 g TYM (Table 8, *P* > 0.05).

### 3.7. Lipid Peroxidation Index

**Figure 2:**
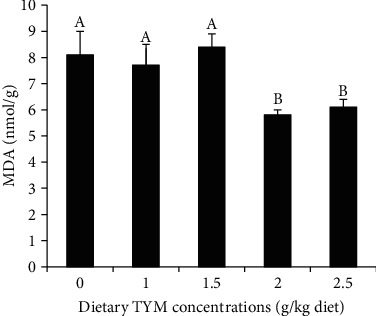
The changes of malondialdehyde (MDA) levels in the rainbow trout, *Oncorhynchus mykiss* over 60 days feeding with dietary levels of thymol (TYM). The differences between the means are indicated as different superscripted letters (*P* < 0.05).

The levels of MDA significantly decreased in fish fed 2-2.5 g TYM compared to other diets (Figure 2, *P* < 0.05). There were no significant differences in MDA levels between the control with fish fed 1-1.5 g TYM (Figure 2, *P* > 0.05).

### 3.8. Gene Expression

**Figure 3:**
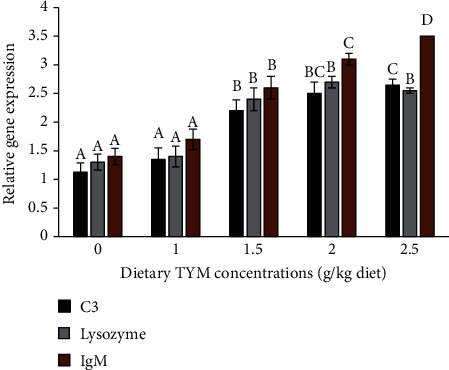
The expression of immune-related genes, complement (C3), Ig, and lysozyme in the rainbow trout, *Oncorhynchus mykiss* over 60 days feeding with dietary levels of thymol (TYM). The differences between the means are indicated as different superscripted letters (*P* < 0.05).

**Figure 4:**
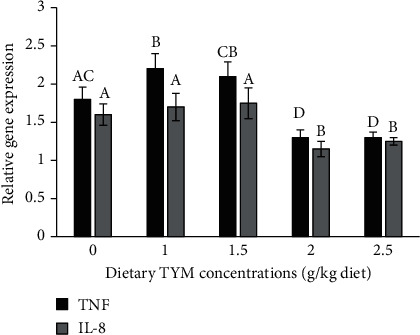
The expression of inflammatory-related genes, TNF-*α* and IL-8 in the rainbow trout, *Oncorhynchus mykiss* over 60 days feeding with dietary levels of thymol (TYM). The differences between the means are indicated as different superscripted letters (*P* < 0.05).

The supplementation of fish with 1.5-2.5 g TYM significantly increased the expression of C3, lysozyme, and Ig genes compared to control (Figure 3, *P* < 0.05). The expression of inflammatory-related genes (TNF-*α* and IL-8) significantly decreased in fish supplemented with 1.5-2.5 g TYM compared to other groups (Figure 4, *P* < 0.05).

### 3.9. Bacterial Challenge

**Figure 5:**
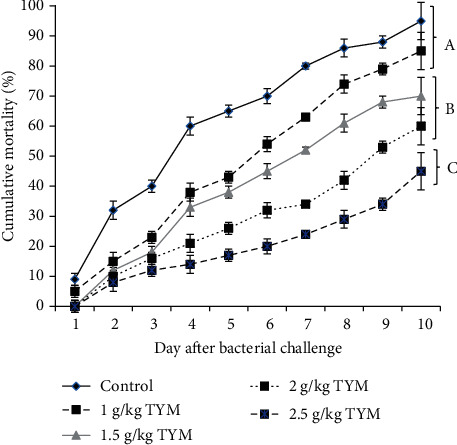
Cumulative mortality of rainbow trout, *Oncorhynchus mykiss* (*n* = 10/tank) fed different concentration of TYM in the diet throughout 10-day challenge with *Streptococcus iniae*. Significant differences are shown with different lower-case letters (*P* < 0.05). Data: Mean ± SD.

After 10 days bacterial challenge, the fish supplemented with 2-2.5 g TYM significantly showed lower mortality rate compared to other experimental groups (Figure 5, *P* < 0.05). The highest mortality rate were observed in control and fish fed 1 g TYM (Figure 5, *P* < 0.05).

## 4. Discussion

Antibiotics are widely used to control bacterial pathogens in aquaculture. Although the bacterial infections can be controlled by antibiotics, their continued use weakens the immune system and may create bacterial resistance strains [2]. Introducing an environmentally and human health friendly alternative to antibiotics could be important. In this way, herbal products and their derivatives have been shown to have high performance [63]. In the present study, we used thymol (TYM) as dietary supplement for rainbow trout. The growth performance improved in response to TYM, as the FW, WG %, and SGR increased in fish fed 1.5-2.5 ml TYM, and FCR decreased in those fed 2-2.5 g TYM. In addition, the body protein content and the activity of digestive enzymes, lipase, and protease increased in response to 1.5-2.5 g TYM. In line with our results, Morselli et al. [64] observed better growth in grass carp, *Ctenopharyngodon idella* supplemented with 100 mg thymol/kg diet. In the rainbow trout, the growth performance improved in fish fed diets containing thymol-carvacrol [34]. Similar results were found in the same fish, where the fish fed 6 g/kg thymol showed better feed efficiency compared to nonsupplemented individuals [24]. In the study of Abd El-Naby et al. [22], thymol alone or in combination with chitosan nanoparticles significantly improved growth performance, feed, and protein utilization in the Nile tilapia, *Oreochromis niloticus*. However, Hoseini and Yousefi [65] did not observe any changes in the growth performance of rainbow trout after a 60-day feeding with a thymol-containing diet (5, 10, and 20 g/kg diet) between experimental treatments. Similar results were observed in the Nile tilapia, where 500 ppm thymol had no effect on the fish growth [66]. Therefore, the effect of thymol on growth may be different depending on dietary levels, fish species, and experiment duration and conditions. In this study, the improved growth performance in the supplemented fish may be due to prompting effects TYM on digestive enzymes, feed utilization, and health status of the fish, as the digestive enzyme activity, the immune and antioxidant components elevated in the fish fed 1.5-2.5 g TYM. According to results, dietary TYM (mostly 1.5-2.5 g/kg diet), prompted the antioxidant and immune system by stimulating the liver antioxidant enzymes (GPx, SOD, and CAT) and immune components of blood (C3, total Ig, protein content, lysozyme, and bactericidal activities) and mucus (Ig, protein content, lysozyme, protease, ALP, and bactericidal activities). Additionally, TYM at dietary levels of 1.5-2.5 g/kg diet upregulated the expression of immune-related genes (C3, lysozyme, and Ig). As a component of humoral defense, Ig immunizes fish through opsonization of pathogens and also neutralization of toxic molecules [67, 68]. Furthermore, the proteins of complement system are involved in both nonspecific and specific immunity by opsonization of pathogens and activating inflammatory reactions [69]. The blood protein content can also be an indicator of the immune status of fish, because it includes antibodies and albumin [70]. Alkaline phosphatase is a mucosal enzyme with antibacterial and hydrolytic activities. Therefore, the elevated levels of ALP in the TYM-supplemented fish may indicate an improvement in immune status [71]. Proteases are a group of fish mucus enzymes with catalytic function on the peptidoglycan layer of bacteria [72]. Enzymatic and nonenzymatic components of antioxidant system are involved in fish immunity through neutralizing free radicals, reducing oxidative stress [16]. SOD catalyzes the dismutation of the superoxide anion (O^2−^) to molecular oxygen and H_2_O_2_. Finally, the generated H_2_O_2_ is eliminated by CAT and GPx action [73].

The results of the present study were in line with other studies. The supplementation of seabream, *Sparus aurata*, with TYM decreased the growth capacity of pathogenic bacteria in the mucus [74]. Dietary TYM enhanced the immune system of Nile tilapia by reducing oxidative stress and by increasing lysozyme activity and Ig levels in the serum [23]. Giannenas et al. [24] indicated that dietary thymol alone or in combination with carvacrol is capable to ameliorate oxidative stress in the rainbow trout and stimulate liver CAT activity and lysozyme and complement activities in serum. The supplementation of Northern snakehead, *Channa argus*, with 300 and 450 mg/kg thymol significantly ameliorated oxidative stress and stimulated immune components (Ig levels, serum acid phosphatase, ALP, complement, and lysozyme activities) in serum, enhanced the activity of antioxidant enzymes (GPx, SOD, and CAT) and increased the resistance to *Aeromonas veronii* infection [13]. In the nile tilapia, a dietary combination of thymol and chitosan nanoparticles stimulated the CAT activity in liver and kidney tissues [22]. In grass carpuse of 100-300 mg/kg feed thymol enhanced the activity of SOD and GPx in liver and ameliorated oxidative stress [75]. The results of the present study were in line with the previous studies, suggesting a potential immune-promoting function for TYM, which may be associated with its stimulating effects on humoral innate immune components, immune-related gene expressions, and antioxidant defense system.

Fish hematology profile is known as an indicator of fish overall health status, which may change in relation to environmental, nutritional, and physiological conditions [31].

In the present study, dietary TYM increased the values of RBC, Hct, and Hb, which may enhance the capacity of oxygen transportation in blood and thus improve the fish health and welfare. In addition, the WBC levels elevated in TYM supplemented the fish, suggesting an immunogenic function for TYM, as previously reported by Ahmadifar et al. [34]. In agreement with our results, Abd El-Naby et al. [22] reported increases in lymphocytes count, Hct, MCV, and MCHC in the Nile tilapia supplemented with TYM.

Cytokines are the signaling and regulating molecules of both innate and fish immune system involved in inflammatory reactions and phagocytic activities [27, 76, 77]. In this study, TYM at dietary levels of 2-2.5 g/kg diet downregulated the expression of inflammatory-related genes (TNF*-α* and IL-8), which this result may be associated with the anti-inflammatory function of TYM, as demonstrated previously in other studies [10, 30]. Kong et al. [13] observed the upregulation of anti-inflammatory (IL-10 and TGF*-β*) and downregulation of inflammatory (HSP70, TNF-*α*, IL-1*β*, and IL-8) genes, following supplementation of the northern snakehead with 300-400 mg thymol/kg feed, which attributed to anti-inflammatory function of thymol.

In this study, the mortality rate was lower in fish fed 2-2.5 g TYM compared to other groups after the bacterial challenge, which clearly indicates the antibacterial properties of TYM. The antibacterial properties of thymol have been previously reported in an *in vitro* study by Heo et al. [78], where TYM efficiently inhibited the growth of the fish pathogens including *Vibrio vulnificus*, *V. parahaemolyticus*, and *V. Anguillarum, Aeromonas salmonicida*, *A. Hydrophila*, and *Edwardsiella tarda*. Also, in the study of Morselli et al. [64], the supplementation of the grass carp with thymol significantly increased the survival rate of the fish by 62.5% following challenge with *Aeromonas hydrophila*.

## 5. Conclusion

In conclusion, the results of the present study suggest an optimum dietary level of 1.5-2.5 g/kg diet for TYM, improving the growth performance, immunity, and resistance against *Streptococcus iniae* infection in the rainbow trout.

## Figures and Tables

**Table 1:** Dietary formulation (g/kg) and proximate composition of the basal diet [39].

Ingredients	(g/kg)	Ingredients	(g/kg)
Fish meal	350	L-carnithine	0.3
Corn gluten	175	Salt	7.5
Wheat flour	180	Vitamin C	0.2
Soybean meal	130	Proximate composition of ingredients	%
Rice bran	36	Dry matter	**91**
Fish oil	23	Crude protein	40
Sunflower oil	48	Crude lipid	15
^1^Vitamin	15	Ash	9
^2^Mineral premix	15	Phosphorous	1.2
Molasses	20	Fiber	3

^1^Vitamin contains amounts per kg of feed: retinol acetate (a): 10,000 IU; Cholecalciferol (D3): 6000 IU; DL-a-tocopheryl acetate (e): 600 mg; menadione sodium bisulfite (K3): 15 mg; L-ascorbic acid (c): 5400 mg; Dbiotin (H2): 2.4 mg; thiamin mononitrate (B1): 45 mg; riboflavin (B2): 75 mg; calcium D-pantothenate (B3): 7200 mg; niacin amide (B5): 135 mg; pyridoxine hydrochloride (B6): 45 mg; folic acid (B9): 24 mg; cyanocobalamin (B12): 120 mg; ^2^Mineral premix (mg/kg): Fe: 60 mg; Cu: 9 mg; Co: 0.7 mg; Se: 0.75 mg; Zn: 90 mg; Mn: 39 mg; I: 3 mg; cholinechloride: 150,000 mg.

**Table 2:** The sequences of the primers used for the real-time PCR in this study.

Genes	Primers	Primer sequences	ID number
Lysozyme	Forward	TGCCTGTCAAAATGGGAGTC	NM_001124716.1
	Reverse	CAGCGGATACCACAGACGTT	

Complement (C3)	Forward	GAGATGGCCTCCAAGAAGATAGAA	L24433.1
	Reverse	ACCGCATGTACGCATCATCA	

TNF-*α*	Forward	GGGGACAAACTGTGGACTGA	AJ277604 AJ401377
	Reverse	GAAGTTCTTGCCCTGCTCTG	

IL-8	Forward	CACAGACAGAGAAGGAAGGAAAG	NM_001124362.1
	Reverse	TGCTCATCTTGGGGTTACAGA	

*β*-Actine	Forward	GGACTTTGAGCAGGAGATGG	U04616.1
	Reverse	ATGATGGAGTTGTAGGTGGTCT	

Ig	Forward	AAAGCCTACAAGAGGGAGACCGAT	NM_001124235.1
	Reverse	AGAGTTATGAGGAAGAGTATGATGAAGGTG	

**Table 3:** The growth and survival (Mean ± SD) of rainbow trout, *Oncorhynchus mykiss* over 60 days feeding with dietary levels of thymol (TYM). For each parameter, the differences between the means are indicated as different superscripted letters (*P* < 0.05).

Parameters	Thymol concentrations (g/kg diet)					*P* values
	Nonsupplemented	1	1.5	2	2.5	
Initial weight (g)	35.8 ± 4.3	36.4 ± 3.2	37.3 ± 5.2	34.7 ± 4.6	35.5 ± 6.5	0.31
Final weight (g)	52.1 ± 5.3^a^	55.9 ± 3.7^a^	65.2 ± 4.4^b^	69.2 ± 4.3^b^	73.1 ± 6.1^b^	0.01
Weight gain (%)	45.5 ± 9.1^a^	53.57 ± 10.3^a^	74.7 ± 9.5^b^	99.4 ± 10.8^c^	105.9 ± 11.5^c^	0.006
Specific growth rate (%/d)	0.63 ± 0.11^a^	0.71 ± 0.08^a^	0.93 ± 0.11^b^	1.15 ± 0.13^bc^	1.21 ± 0.11^c^	0.014
Feed conversion ratio	2.1 ± 0.25^a^	2.2 ± 0.3^a^	1.9 ± 0.15^a^	1.4 ± 0.15^c^	1.45 ± 0.2^c^	0.022
Survival (%)	93.4 ± 2.3	94.4 ± 3.2	96.2 ± 1.5	95.2 ± 2.1	95.2 ± 2.2	0.032
Feed intake (kg)	53.2 ± 1.1^a^	55.1 ± 2.3^ab^	58.1 ± 2.6^b^	63.5 ± 2.4^c^	65.1 ± 1.7^c^	0.03

**Table 4:** The body composition (%) of rainbow trout, *Oncorhynchus mykiss* (*n* = 15) over 60 days feeding with dietary levels of thymol (TYM). For each parameter, the differences between the means (Mean ± SD) are indicated as different superscripted letters (*P* < 0.05).

Parameters	Thymol concentrations (g/kg diet)					*P* values
	Nonsupplemented	1	1.5	2	2.5	
Protein	12.2 ± 0.6^a^	13.1 ± 0.5^a^	14.5 ± 0.3^b^	14.8 ± 0.4^b^	15.3 ± 0.3^b^	0.04
Lipid	10.9 ± 0.7	11.4 ± 0.5	12.1 ± 0.6	12.4 ± 0.3	12.3 ± 0.2	0.25
Moisture	59.2 ± 2.3	60.1 ± 2.4	61.4 ± 2.5	60.3 ± 1.8	60.6 ± 1.3	0.11
Ash	6.2 ± 0.3	6.5 ± 0.5	5.8 ± 0.6	5.7 ± 0.4	5.8 ± 0.5	0.33

**Table 5:** The activity of digestive enzymes (IU/mg protein) in rainbow trout, *Oncorhynchus mykiss* (*n* = 15) over 60 days feeding with dietary levels of thymol (TYM). For each parameter, the differences between the means (Mean ± SD) are indicated as different superscripted letters (*P* < 0.05).

Parameters	Thymol concentrations (g/kg diet)					*P* values
	Nonsupplemented	1	1.5	2	2.5	
Amylase	0.56 ± 0.15	0.59 ± 0.13	0.68 ± 0.07	0.65 ± 0.09	0.72 ± 0.08	0.15
Protease	2.34 ± 0.05^c^	2.28 ± 0.09^bc^	3.72 ± 0.07^b^	3.9 ± 0.11^b^	4.4 ± 0.15^c^	0.02
Lipase	0.77 ± 0.03^b^	0.95 ± 0.03^b^	1.40 ± 0.25^b^	1.63 ± 0.19^bc^	1.98 ± 0.18^c^	0.01

**Table 6:** The immune components of blood and mucus (Mean ± SD) in rainbow trout, *Oncorhynchus mykiss* (*n* = 15) over 60 days feeding with dietary levels of thymol (TYM). For each parameter, the differences between the means are indicated as different superscripted letters (*P* < 0.05).

	Thymol concentrations (g/kg diet)					*P* values
	Nonsupplemented	1	1.5	2	2.5	
Plasma immune components						
Lysozyme activity (IU/ml)	66.4 ± 10.3^a^	70.6 ± 11.1^ab^	95.8 ± 12.5^b^	121.7 ± 9.2^c^	130.3 ± 10.2^c^	0.011
C3 activity (IU/ml)	19.11 ± 6.3^a^	21.23 ± 4.5^a^	28.1 ± 3.3^ab^	32.6 ± 5.4^b^	38.6 ± 6.3^b^	0.021
Ig (mg/ml)	3.35 ± 0.8^a^	4.71 ± 0.6^a^	6.8 ± 1.1^b^	8.9 ± 1.11^b^	9.6 ± 2.12^b^	0.03
Bactericidal activity (No. of. CFUs)	122.5 ± 10.3^a^	115.5 ± 9.1^a^	135.6 ± 12.1^b^	152.6 ± 10.2^b^	149.3 ± 8.3^b^	0.01
Total protein (g/dl)	3.4 ± 0.5^a^	4.1 ± 0.52^b^	5.1 ± 0.21^b^	7.2 ± 0.18^c^	6.5 ± 0.31^c^	0.013

Mucosal immune components						
Lysozyme activity (IU/ml)	25.3 ± 5.2^a^	30.5 ± 4.1^a^	45.7 ± 5.2^b^	50.6 ± 6.1^b^	65.4 ± 4.2^c^	0.02
Ig (mg/dl)	1.4 ± 1.1^a^	1.3 ± 1.2^a^	4.5 ± 1.3^b^	5.3 ± 1.2^b^	8.2 ± 1.2^c^	0.014
Protease activity (IU/ml)	6.3 ± 1.4^a^	8.1 ± 2.1^ab^	11.3 ± 2.2^bc^	14.4 ± 1.4^bc^	14.6 ± 1.5^c^	0.01
Alkaline phosphatase activity (IU/ml)	1.25 ± 0.4^a^	1.34 ± 0.5^a^	2.55 ± 0.32^b^	3.3 ± 0.17^c^	4.58 ± 0.21^d^	0.022

∗ C3: complement C3, Ig: total immunoglobulin.

**Table 7:** The hematological alternations (Mean ± SD) in rainbow trout, *Oncorhynchus mykiss* (*n* = 15) over 60 days feeding with dietary levels of thymol (TYM). For each parameter, the differences between the means are indicated as different superscripted letters (*P* < 0.05).

Parameters	Thymol concentrations (g/kg diet)					*P* values
	Nonsupplemented	1	1.5	2	2.5	
RBC (×10 ^6^/*μ*l)	1.5 ± 0.12^a^	1.7 ± 0.21^a^	1.45 ± 0.12^a^	2.5 ± 0.1^b^	2.8 ± 0.13^c^	0.01
WBC (×10^3^/*μ*l)	5.5 ± 1.2^a^	5.8 ± 1.4^a^	7.2 ± 1.8^ab^	10.6 ± 2.3^b^	11.5 ± 2.5^c^	0.015
Hct (%)	24.5 ± 2.4^a^	25.4 ± 1.8^a^	27.9 ± 2.6^a^	33.2 ± 2.4^b^	35.1 ± 2.7^b^	0.008
Hb (g/dl)	1.99 ± 0.15^a^	2.02 ± 0.51^a^	2.1 ± 0.13^a^	3.41 ± 0.11^b^	3.6 ± 0.21^b^	0.011
MCV (fl)	163.3 ± 10.3^a^	149.4 ± 11.3^ac^	192.4 ± 10.1^b^	132.8 ± 14.1^c^	125.3 ± 13.4^c^	0.02
MCH (pg)	13.2 ± 2.2	11.88 ± 1.7	14.4 ± 2.2	13.64 ± 2.1	12.85 ± 2.2	0.035
MCHC (%)	0.81 ± 0.12^a^	0.79 ± 0.13^a^	0.75 ± 0.1^a^	1.02 ± 0.12^b^	1.03 ± 0.13^b^	0.002

RBC: red blood cell, WBC: white blood cell, Hct: hematocrit, Hb: hemoglobin, MCV: mean corpuscular volume, MCH: mean corpuscular hemoglobin, MCHC: mean corpuscular hemoglobin concentration.

**Table 8:** The liver antioxidant changes (IU/mg) in rainbow trout, *Oncorhynchus mykiss* (*n* = 15) over 60 days feeding with dietary levels of thymol (TYM). For each parameter, the differences between the means (Mean ± SD) are indicated as different superscripted letters (*P* < 0.05).

Parameters	Thymol concentrations (g/kg diet)					*P* values
	Nonsupplemented	1	1.5	2	2.5	
SOD	4.4 ± 1.1^a^	5.1 ± 1.2^a^	8.1 ± 1.7^b^	9.3 ± 2.13^b^	11.5 ± 2.14^b^	0.02
CAT	5.2 ± 1.3^a^	7.9 ± 1.55^b^	8.1 ± 1.7^b^	11.2 ± 1.3^c^	12.3 ± 1.2^c^	0.01
GPx	3.1 ± 0.08^a^	4.11 ± 0.1^a^	7.8 ± 0.13^b^	8.5 ± 0.1^b^	9.7 ± 0.13^b^	0.04

∗ SOD: superoxide dismutase, CAT: catalase, GPx: glutathione peroxidase.

## Data Availability

The datasets generated during and/or analysed during the current study are available from the corresponding author on reasonable request.
